# PAD: a UK Perspective - Contemplating Change is Challenging!

**DOI:** 10.1192/j.eurpsy.2024.117

**Published:** 2024-08-27

**Authors:** J. Wise

**Affiliations:** CNWL, London, United Kingdom

## Abstract

**Abstract:**

Physician assisted dying is not new, neither historically, nor globally. What has changed in the UK however, is the perspective of society. In the UK, the British Medical Association is both a union and a professional organisation representing doctors and liaising with governments departments in matters of healthcare. As with various specialties within medicine, there are those in favour of change and those against. There are matters on which there is common ground, and a consensus of experts has identified principles, which, if legislation is to change, would be sensible to follow. A profession has united around the idea that if change is coming, it is better to inform the debate proactively and ensure that the interest of patients and doctors are promoted.  This session will look at how potential change in the UK has been approached and hopefully well managed.’

**Disclosure of Interest:**

None Declared

